# Update on Obesity and Its Relationship to Atherosclerotic Cardiovascular Disease and Associated Risk Factors

**DOI:** 10.3390/jcm15124430

**Published:** 2026-06-08

**Authors:** Yaser Ahmad, Raj Wasan, Jordan D. Perchik, Navin C. Nanda

**Affiliations:** 1 Department of Internal Medicine, University of Alabama at Birmingham, Birmingham, AL 35294, USA; 2 College of William and Mary, Williamsburg, VA 23185, USA; 3 Department of Radiology, University of Alabama at Birmingham, Birmingham, AL 35294, USA; 4 Division of Cardiology, University of Alabama at Birmingham, Birmingham, AL 35294, USA

**Keywords:** obesity, lifestyle interventions, surgery, pharmacologic therapy, imaging, anthropometric measures, cardiovascular disease, GLP-1 drugs

## Abstract

**Background/Objectives**: Obesity is a multifactorial chronic condition characterized by the accumulation of excess adiposity and complex interplay between intrinsic and extrinsic factors. It is an increasingly common condition, closely implicated with the incidence and progression of cardiovascular disease and its risk factors. This narrative review synthesizes and summarizes recent evidence on obesity, with a focus on the diagnosis of obesity, an exploration of both visceral and subcutaneous adipose tissue, available interventions for obesity ranging from dietary modifications to novel anti-obesity medications, and key associations with obesity and cardiovascular diseases. This review is distinct in its integrated focus on obesity definition and diagnosis, imaging modalities, the latest non-pharmacologic and pharmacologic interventions, and also the interplay between obesity and certain cardiovascular conditions as well as their risk factors. **Results**: The diagnosis of obesity has been evolving with the incorporation of anthropometric measurements and imaging modalities rather than simply the body mass index. There is a wide array of contributors to obesity including genetic factors, behavior, hormonal regulators, the brain–gut axis, and psychosocial stressors. Anti-obesity medications have been evolving rapidly, with current emphasis on glucagon-like peptide 1 receptor agonists. Obesity is closely implicated in cardiovascular conditions such as atherosclerotic disease, heart failure, atrial fibrillation, and hypertension as well as related risk factors such as diabetes mellitus, chronic kidney disease and sleep apnea. **Conclusions**: Obesity is a widely prevalent, chronic, and complex disease. The use of a variety of anthropometric measurements can help risk-stratify individuals. Imaging techniques are also helpful in evaluating body fat. Evaluating individuals from a holistic perspective is imperative to appreciate the various contributors to obesity. There are a variety of interventions available for obesity management including lifestyle interventions, bariatric surgery, and pharmacologic therapy. Notably, obesity is closely tied with cardiovascular diseases and recent pharmacologic anti-obesity agents may mitigate cardiovascular risk.

## 1. Introduction

Obesity is a multifactorial chronic condition that is characterized by excessive body fat accumulation, with complex interactions between genetics, environment, and behavior. Obesity has detrimental effects on numerous organs, and the majority of deaths in patients with high body mass index (BMI) are due to cardiovascular disease (CVD) [1]. It is a major problem since it affects around a billion individuals worldwide [2]. The aim of this review is to provide a comprehensive update on obesity with emphasis on the definition and diagnosis of obesity, pharmacological and non-pharmacological interventions for increased body weight, and also the relationship between obesity and cardiovascular disease as well as associated risk factors. We aim to highlight the various anthropometric measurements available for classifying obesity, explore the role of point-of-care ultrasound in assessing body fat, offer a succinct summary of current anti-obesity medications that can aid in clinician decision-making, and briefly discuss the future of anti-obesity management.

## 2. Methods

The current review was conducted using a comprehensive search that was conducted using the PubMed and MedLine databases on all the relevant literature within the past five years. The keywords used during the search were “obesity,” “cardiovascular disease,” “diagnosis,” and “treatment.”

## 3. Diagnosing Obesity

### 3.1. Defining Obesity

The World Health Organization (WHO) report published recently in 2025 uses BMI to classify individuals as overweight or obese. BMI is calculated by dividing an adult’s weight in kilograms by their height in meters squared. The WHO defined overweight as having a BMI greater than 25 kg/m^2^ in adults, while obesity is defined as having a BMI greater than 30 kg/m^2^ in adults. Obesity can be broken down into the following classes: Class 1 (BMI 30 to <35 kg/m^2^), Class 2 (BMI 35 to <40 kg/m^2^), and Class 3 (BMI ≥ 40 kg/m^2^). In 2025, The Lancet Diabetes and Endocrinology released an expert consensus statement addressing the definition of obesity. They defined preclinical obesity as a state of excess adiposity without evidence of tissue or organ dysfunction. They recommended measuring excess adiposity either directly using modalities such as dual-energy X-ray absorptiometry (DEXA) or bioimpedance analysis, if possible, or through anthropometric measurements such as waist circumference or the waist-to-height ratio, described below. While BMI should be used only as a surrogate marker according to their recommendation, it can be assumed that individuals with a BMI greater than 40 kg/m^2^ have excess adiposity. They define clinical obesity as the presence of reduced tissue or organ function as a result of obesity and/or significant limitations in basic activities of daily living due to the effects of obesity [3].

BMI is a quick and simple measurement that has been widely used and allows for a measurement of general adiposity, but Lancet’s expert consensus statement describes BMI as a method for assessing health risk at the population level rather than a method for assessing the health risk of an individual [3]. Other shortcomings of BMI include the fact that it cannot distinguish between visceral and subcutaneous adiposity. Unlike subcutaneous adipose tissue (SAT), which represents 80% of fat in our body, visceral adipose tissue (VAT) consists of fat within and surrounding abdominal organs such as the liver, pancreas and kidneys as well as the omentum and mesenteric folds. VAT may increase with age and factors such as heavy alcohol consumption, stress, and poor sleep [4]. Also, there is evidence to suggest that compared to SAT, VAT is more metabolically active and linked to insulin resistance, thus conferring more cardiometabolic risk [5,6]. Waist circumference (WC), which is simple to measure, correlates with VAT and hence can be used as a surrogate for visceral adiposity. Higher WC is also associated with increased mortality [7]. WC is generally taken at the midpoint between the lowest margin of the last palpable rib and top of the iliac crest, and normal values are 102 cm (40.2 in) or less in men and 88 cm (34.6 in) or less in women.

The waist-to-hip ratio can also be used to assess obesity and is calculated by dividing WC by hip circumference (both in the same units). It correlates well with VAT. An increased waist-to-hip ratio is associated with cardiovascular disease and metabolic disorders [8].

Another simple measurement is the waist-to-height ratio (WHtR) which is calculated by dividing WC by height (both in the same units) and also correlates with central obesity (truncal obesity, practically similar to VAT) [9,10]. A normal WHtR is <0.5 in both men and women [8]. In clinical practice, both the anthropometric measurements of WC and the WHtR are best interpreted in the context of BMI. The WHtR may be superior to WC in some ways. For instance, if two individuals were to have identical WCs, but one individual was taller than the other, the WC alone would confer identical cardiovascular risk in both individuals. However, this is not true, as the percentage body fat would differ between the two individuals, as a result of their different heights. As such, the WHtR provides a more complete representation of cardiovascular risk and has even been shown to be a superior discriminator for certain risk factors such as hypertension and diabetes [9,11]. A 2026 study demonstrated that the WHtR can accurately predict the risk of hypertension in the adult and youth population. In this study, excess fat was defined as a WHtR of greater than or equal to 0.53 in males and 0.54 in females [12].

A Body Shape Index (ABSI), another measurement, is calculated based on WC and is adjusted for height and weight [13,14]. A high ABSI measure corresponds to a more central concentration of body volume (visceral fat). ABSI has been shown to predict mortality across age, sex, and weight, which can be attributed to its superior correspondence to visceral fat compared to other anthropometric measures [14]. In addition, it is possible that individuals with a high ABSI have less lean muscle mass, which is thought to have a negative correlation with mortality risk.

Relative fat mass (RFM), another index, is a measure for estimating body fat percentage using waist circumference and height. The calculation for RFM is as follows:

$$Relative\;fat\;mass=64-\left(20\times \frac{height}{waist}\right)+\left(12\times sex\right),\;$$

where sex=0 for males and 1 for females

Average RFM values are 25% in males and 35% in females [15]. Obesity is defined as an RFM cutoff of 30% in males and 40% in females [16]. RFM is a strong predictor of obesity-related conditions such as heart failure and type 2 diabetes mellitus (T2DM) [17]. A summary of these indices is listed in Table 1.

The specific cutoffs for BMI have been extensively discussed in specific ethnic groups. For instance, there has been concern that the association between BMI and cardiovascular disease is different in Asian populations [18]. In particular, there is a high proportion of Asians with lower BMI than traditional cutoffs for obesity that exhibit high rates of T2DM or cardiovascular disease. As such, the WHO concluded that a BMI of 23 kg/m^2^ may confer “increased risk” for Asians and that a BMI of greater than 27.5 kg/m^2^ could be considered as “high-risk” [18]. In a population-based cohort study, Caleyachetty et al. utilized electronic health records in England to derive BMI cutoffs in specific ethnic groups that were equivalent to the age- and sex-adjusted incidence of T2DM in White populations with a BMI of 30 kg/m^2^. They stated that equivalent BMI cutoffs were 23.9 kg/m^2^ in South Asians, 28.1 kg/m^2^ in Black individuals, 26.9 kg/m^2^ in Chinese populations, and 26.6 kg/m^2^ in Arab populations [19]. Thus, South Asians have a higher risk of developing insulin resistance, despite lower BMIs, compared to White populations [20]. Interestingly, South Asians have a unique pattern of fat deposition as well. Compared to White individuals with an equivalent BMI, South Asians have a higher body fat percentage and increased abdominal adiposity.

**Table 1:** Indices and modalities for assessing obesity.

Index	Method of Calculation	Classification	Advantages	Limitations
Body mass index (BMI)	Metric: Weight in kilograms divided by height in meters squared. Imperial: Weight in pounds divided by height in inches squared, multiplied by 703.	Class 1 (BMI 30 to <35 kg/m^2^), Class 2 (BMI 35 to <40 kg/m^2^), and Class 3 (BMI ≥ 40 kg/m^2^).	Useful in screening and can provide useful information at the population level.	Unable to quantify an individual’s body fat amount or adipose tissue distribution [21]. Unable to differentiate between subcutaneous adipose tissue (SAT) and visceral adipose tissue (VAT) [22].
Waist circumference	No consensus method. The World Health Organization recommends the measurement be made at the midpoint between the lower margin of the last palpable rib and the top of the iliac crest [23]. The National Institutes of Health recommends the measurement be made immediately above the iliac crest [24].	No universally adopted cutoffs. For men, 102 cm (40.2 inches) and 88 (34.6 in) cm for women (National Heart Lung and Blood Institute) [25].	Strong relationship with cardiovascular risk factors. Strong correlation with visceral/intra-abdominal fat [26].	Measurements may not be consistent (dependent on measuring tape, posture, phase of respiration at time of measurement, etc.).
Waist-to-hip ratio	Ratio between waist circumference (measured at the narrowest point between the ribs and hips) and hip circumference (measured at the widest part of the buttocks) in the same unit [8,27].	Men—below 0.90 is normal. Women—below 0.85 is normal [8].	Strong association with overall death, cardiovascular disease (CVD), and type 2 diabetes mellitus (T2DM) [28]. Correlates with VAT [8].	Limited utility when both waist and hip circumferences change due to this index being a ratio.
Waist-to-height ratio	Ratio between waist circumference (measured at the narrowest point between the ribs and hips) and height in the same unit [8,27].	Greater than 0.5 indicates increased metabolic risk [9].	Allows for assessment of central adiposity/VAT. Robust indicator for hypertension, T2DM, and dyslipidemia [11].	Lack of universal method for measuring waist circumference.
Relative fat mass index	64 − (20 x (height/waist)) + (12 x sex), where sex = 0 for males and 1 for females.	Obesity is defined as >30% in males and >40% in females.	More accurate calculation of total body fat percentage, strong correlation with heart failure, T2DM, CVD, atrial fibrillation [29].	Reliance on accurate waist measurements; further investigation required for optimal cutoffs for obesity.
Ultrasound	Can be used to measure SAT and VAT thickness.	Lack of standardized cutoffs.	Valid and fast assessment of SAT and VAT. No ionizing radiation.	Inherent artifacts may be encountered with image acquisition. Dependent on operator. Techniques not standardized.
Magnetic resonance imaging	Assessment of body compartments and muscle mass [30]. Can provide volumetric assessments.	No universally adopted cutoffs.	High accuracy, lack of radiation. Considered the gold standard for SAT and VAT measurement.	Not available at all institutions, and data assessment may be difficult for novices.
Computed tomography (CT)	Can accurately quantify VAT and SAT. Can be used to calculate the VAT index, which is the visceral fat area divided by height in meters squared.	VAT index greater than 38.7 cm^2^/m^2^ in males and 24.9 cm^2^/m^2^ is associated with increased metabolic risk [31].	High accuracy, can be used to risk-stratify individuals for metabolic disease.	Limited access, radiation.
Dual-energy X-ray absorptiometry (DEXA)	Measures whole body and regional fat distribution. Can measure VAT as well as percent body fat.	Lack of standardized cutoffs. Proposed cutoffs include 700 g in women younger than 40 yrs; 800 g in women older than 40 yrs; 1000 g in men younger than 40 yrs; and 1200 g in men older than 40 yrs [32]. There are no universally accepted cutoffs for percent body fat when calculated through DEXA. However, previous work has suggested that obesity could be defined as greater than 30% in men and 42% in women [33].	Relatively low amount of X-ray radiation, strong correlation to CT-measured VAT [34].	Although relatively little, this imaging modality still uses radiation. Systematic variations between software [35].
Bioelectrical impedance analysis (BIA)	Sends a low-level electrical current, and based on the resistance encountered by fat tissue, it can be used to calculate body fat percentage [36].	Lack of standardized cutoffs, although in a previous study using BIA in women, a cutoff of 35% body fat was used to define obesity [37].	Noninvasive, quick, easy to perform [38].	Severe obesity may overestimate body fat [39].

### 3.2. Ultrasound-Guided Assessment

Traditionally, magnetic resonance imaging (MRI) and computed tomography (CT) have been used to assess SAT and VAT. SAT is defined as the distance between the front edge of the abdominal muscles and skin, while VAT is the distance between the posterior edge of the abdominal muscles and front of the lumbar spine [40]. In a 2025 study, ultrasound was used to assess abdominal fat distribution. Ultrasound was found to correlate quite well with MRI-measured VAT thickness. The VAT/SAT ratio (thickness) is associated with CVD risk [41], although there are no universally accepted cutoffs for this ratio. Previous work has demonstrated a correlation between anthropometric indices and ultrasound-measured VAT thickness. Ultrasound-measured VAT thickness has been associated with metabolic risk factors such as fasting blood sugar and triglyceride levels [42].

SAT and VAT measurement can feasibly be integrated into an abdominal ultrasound examination or performed on a point-of-care basis. SAT and VAT can be measured on ultrasound with the patient in the supine position using a standardized midline abdominal approach, most commonly in the supraumbilical region, although there is variability in the exact measurement site. In general, maximal SAT thickness is often obtained with the probe in the transverse position and closer to the xyphoid process, whereas VAT measurements can be obtained transversely or longitudinally and are frequently performed slightly more caudally, where intra-abdominal fat is more prominent [43,44]. A high-frequency linear transducer (typically ~7–15 MHz) is preferred for SAT assessment due to its superior near-field resolution, allowing for precise measurement from the inner border of the skin to the linea alba or anterior rectus fascia [43,44]. The linea alba is the midline thin and echogenic seam between the bellies of the left and right rectus abdominus muscles. In contrast, VAT is better evaluated using a lower-frequency curvilinear (convex) transducer (typically ~2–5 MHz), which provides greater penetration to visualize deeper retroperitoneal structures such as the aorta [43,44]. Landmarks for VAT measurement can be variable; some protocols define VAT as the distance from either the peritoneum or linea alba to either the anterior aortic wall, posterior aortic wall, or anterior surface of the vertebral body [43,44,45,46]. Care should be taken to minimize probe pressure to avoid the compression of fat layers, and measurements are typically obtained at end-expiration to improve reproducibility. Prior studies have demonstrated that these sonographic indices correlate reasonably well with CT- or MRI-derived abdominal fat distribution, supporting ultrasound as a practical, noninvasive method for estimating central adiposity in clinical practice [43,44,45,46,47,48].

Important limitations and challenges remain. Ultrasound is inherently operator-dependent, and measurements may be influenced by probe pressure, respiratory variation, patient body habitus, and the inconsistent selection of anatomic landmarks or measurement location [43,44,45]. While bowel gas or abdominal distension may impair image quality, ultrasound protocols do not explicitly state that patients must be fasting prior to imaging being performed. The visualization of deep structures such as the aorta may be limited in patients with increased abdominal wall thickness or bowel gas, further affecting reproducibility. A key unresolved issue is the lack of standardization in VAT measurement, resulting in systematic discrepancies in reference values for SAT and VAT measurements across studies [43,44,45,46]. The lack of a standardized technique for VAT measurement is a major stumbling block to the wider adoption of SAT and VAT, and future studies are needed to determine the optimal ultrasound technique and reference standards for SAT and VAT values at the population level.

Despite these challenges, the increasing accessibility of ultrasound in the outpatient setting, performed both in radiology departments and on a point-of-care basis, makes the ultrasonographic assessment of SAT and VAT an attractive target for early diagnosis and intervention for metabolic syndrome. Advancements in opportunistic screening are not limited to ultrasound, as advances in radiology Artificial Intelligence (AI) have made quantitative fat segmentation more feasible at a large scale. Opportunistic screening AI algorithms have the capability to perform autonomous segmentations of visceral and subcutaneous fat and provide a quantitative score to predict the likelihood of metabolic syndrome [49]. Additionally, these algorithms can provide metrics on bone mineral density, skeletal muscle bulk, and aortic calcium burden to generate a more comprehensive assessment of a patient’s health [49]. As these algorithms enter the marketplace, the data generated by opportunistic CT screening tools and ultrasound could serve complementary roles in the early detection of metabolic syndrome.

Notably, while the primary focus of this discussion is the role of point-of-care ultrasound in SAT and VAT assessment, there are other relevant structures that can be measured using ultrasound that hold relevance in terms of metabolic risk. Particularly, attention can be drawn to the various compartments of visceral fat. For instance, perirenal fat thickness may be a surrogate marker for metabolic syndrome [50]. In addition, pre-peritoneal fat thickness has been associated with dyslipidemia, nonalcoholic fatty liver disease, and obesity [51]. VAT is also associated with carotid plaque burden, which can be assessed by ultrasound [52]. Furthermore, ultrasound can help delineate the epicardial adipose tissue (EAT) layer, an anatomic fat layer that sits between the outer surface of the myocardium and visceral pericardium. It is a key metabolic regulator and is implicated in cardiometabolic risk. Standard parasternal long- and short-axis views can be used to visualize the EAT layer. Measurements of the layer are usually taken at end-systole when the heart is contracted. While there are no consensus reference values, the current literature suggests a thickness greater than 7 mm to be abnormal. A more detailed description of the EAT layer and metabolic risk is further provided below [53].

**Figure 1:**
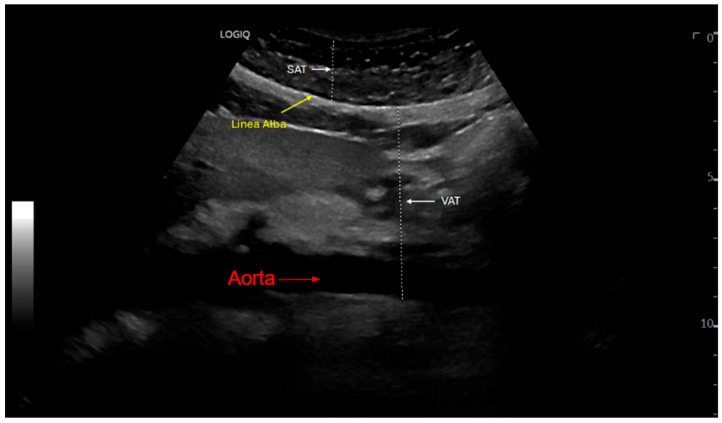
A transabdominal ultrasound image demonstrating subcutaneous and visceral adipose tissue: A transabdominal gray-scale ultrasound image of the midline upper abdomen acquired with a low-frequency (high-depth) ultrasound probe in the supraumbilical region in a longitudinal position. The longitudinal orientation of the aorta (red arrow) demonstrates that this image is acquired in the sagittal orientation. Subcutaneous adipose tissue (SAT) is measured from the deep aspect of the epidermis to the linea alba (the echogenic line marked by the yellow arrow). In this image, visceral adipose tissue (VAT) is measured from the linea alba to the posterior wall of the aorta. Figure courtesy of Jordan Perchik MD.

**Figure 2:**
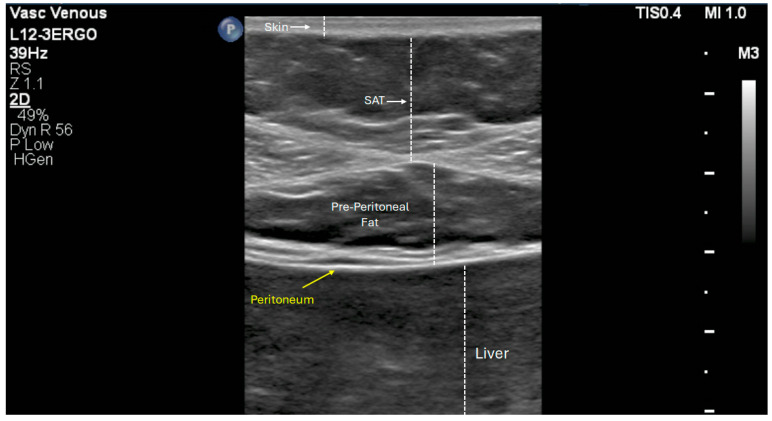
A transabdominal ultrasound image demonstrating pre-peritoneal fat in addition to subcutaneous and visceral adipose tissue: A transabdominal gray-scale ultrasound image of the midline upper abdomen acquired with a high-frequency (low-depth) ultrasound probe in a transverse position. Subcutaneous adipose tissue (SAT) is measured from the deep margin of the skin to the linea alba (yellow arrow). The linea alba can be identified as the bright echogenic tissue connecting the darker striated muscle bellies of the rectus abdominis muscles. Pre-peritoneal fat is the fat pad between the linea alba and the peritoneum (the deeper echogenic linear structure superficial to the liver). Pre-peritoneal fat thickness has also been identified as a potential imaging marker correlating to metabolic-related cardiovascular diseases. Figure courtesy of Jordan Perchik MD.

**Figure 3:**
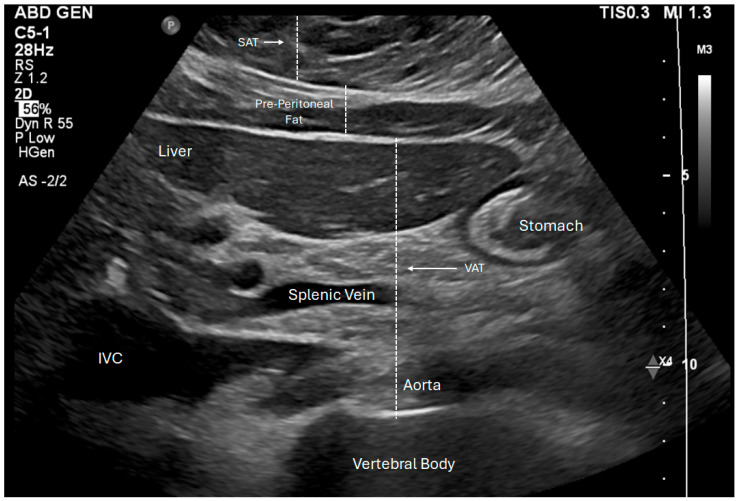
A transabdominal ultrasound image demonstrating regional landmarks: A transabdominal gray-scale ultrasound image of the midline upper abdomen acquired with a low-frequency (high-depth) ultrasound probe in a transverse position. Typical anatomic structures that are seen in the field of view when measuring VAT are annotated, including the liver, stomach, inferior vena cava (IVC), splenic vein, aorta, and vertebral body. In this image, visceral adipose tissue (VAT) is measured from the peritoneum (thin, echogenic, linear structure just superficial to the liver, to the posterior aspect of the aorta), as opposed to the method described in Figure 1. The different methods of VAT measurement highlight the potential pitfalls of comparing reference values or subcutaneous adipose tissue (SAT)/VAT ratios between different studies. Figure courtesy of Jordan Perchik MD.

Figure 1, Figure 2 and Figure 3 display point-of-care ultrasound images with SAT, VAT, and regional landmarks. Notably, Figure 2 also shows pre-peritoneal fat, which is located between the transversalis fascia (lining fascia of the anterolateral abdominal wall) and parietal peritoneum. While measurements of this fat layer may provide similar or complementary information to VAT, further research is needed to elucidate its significance [51].

### 3.3. Computed Tomography and Magnetic Resonance Imaging Assessments

Computed tomography (CT) allows for the direct measurement of adipose tissue from cross-sectional imaging slices. It can be used to measure both SAT and VAT. It is a relatively cumbersome approach and also exposes individuals to radiation [54]. Therefore, the measurement of adipose tissue using CT is especially useful when the images are acquired for other medical indications. While there are no universally accepted reference ranges, a cutoff of 100 cm^2^ for VAT has been suggested for increased cardiovascular risk [55]. Magnetic resonance imaging (MRI) can quantify fat using fat–water imaging techniques, leading to a very detailed assessment of the distribution of fat depots. It can be used to assess both SAT and VAT. Recent advances in imaging techniques have also decreased analysis time. There can also be differences in imaging protocols, which affects the amount of adipose tissue estimated [56]. MRI is considered the gold standard for the assessment of SAT and VAT [57]. There are no universally adopted reference ranges for MRI estimations of SAT or VAT.

### 3.4. Dual-Energy X-Ray Absorptiometry Assessment

Dual-energy X-ray absorptiometry (DEXA) is a common imaging modality used to assess adipose tissue deposits. In a DEXA scan, photons are emitted from an X-ray source. DEXA image acquisition is based on the rate of radiation absorption by tissue in the body. DEXA can be used to determine the extent of SAT and VAT. DEXA can provide information about VAT volume, cross-sectional area, and weight. DEXA-measured VAT correlates well with CT-measured VAT. An advantage of DEXA is that it emits lower levels of radiation compared to CT [58]. DEXA is also more widely available and less costly than CT and MRI. There are no universal cutoffs for measurements obtained by DEXA, but studies have suggested reference ranges. For instance, Meredith-Jones et al. found that the following VAT cutoffs indicated increased cardiometabolic risk: 700 g in women younger than 40 yrs; 800 g in women older than 40 yrs; 1000 g in men younger than 40 yrs; and 1200 g in men older than 40 yrs [32]. A limitation of DEXA is that obesity itself leads to artifacts, which can alter interpretation [59].

## 4. Contributors to Obesity

**Figure 4:**
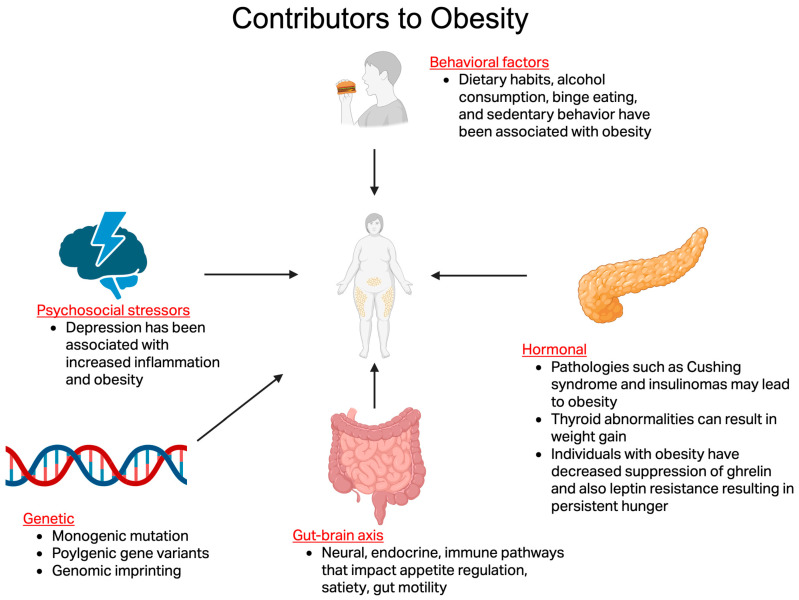
Contributors to obesity. Illustration courtesy of Yaser Ahmad MD.

Figure 4 demonstrates an overview of the contributors to obesity.

### 4.1. Genetic Causes

There are presumed to be genetic causes of obesity. For instance, a risk factor for childhood or adolescent obesity is paternal obesity [60]. Monogenic obesity is characterized by a mutation in a single gene that results in early-onset and severe obesity. It rarely occurs and has high penetrance. However, there is still variable expressivity and incomplete penetrance that affects the phenotype. Greater than 50% of the genes implicated in monogenic obesity act directly within the hypothalamic leptin–melanocortin pathway [61]. In contrast, polygenic obesity is characterized by gene variants that occur in multiple genes, resulting in cumulative effects that precipitate obesity. Polygenic obesity also can be influenced by environmental factors [62]. Genomic imprinting, an epigenetic process, affects gene expression without changing the deoxyribonucleic acid (DNA) sequence and is implicated in certain obesity syndromes such as Prader–Willi syndrome [63]. Interestingly, the heritability of BMI varies across the BMI spectrum, with individuals who are obese or severely obese having greater BMI heritability compared to normal-weight individuals [64].

Advances in genetics have allowed for the development of precision medicine. For instance, in patients with leptin deficiency caused by *LEP* deficiency, recombinant leptin has been a crucial treatment option. In patients with Prader–Willi syndrome, diazoxide choline was approved in 2025 by the United States Food and Drug Administration. This medicine opens adenosine triphosphate (ATP)-sensitive potassium channels and has been shown to improve hyperphagia [61]. In addition, the leptin–melanocortin pathway is a hypothalamic pathway essential for the regulation of weight. Defective genes in this pathway can lead to defective protein products, resulting in genetic obesity. α- and β-melanocyte-stimulating hormones (MSHs) are crucial in this pathway and bind to the melanocortin-4 receptor (MC4R), leading to satiety. Setmelanotide is a medication used to treat genetic obesity. It is an MC4R ligand that helps bypass any genetic defects upstream, described in detail later [65].

### 4.2. Endocrine Abnormalities

Endocrine abnormalities can also contribute to obesity. An insulinoma, an islet cell tumor of the pancreas, can result in rapid weight gain. Patients with insulinoma have excess levels of insulin, which leads to symptoms of hypoglycemia, including but not limited to intense hunger, palpitations, and dizziness. To mitigate these symptoms, patients learn to eat frequent meals, resulting in rapid weight gain [66]. Cushing syndrome, another endocrine pathology, occurs due to prolonged exposure to high levels of cortisol. The clinical features of this syndrome include rapid weight gain and central adiposity accumulation [67]. In addition, thyroid hormone plays a major role in metabolism and can upregulate the basal metabolic rate. Hypothyroid patients tend to exhibit weight gain. Hypothyroidism is characterized by glycosaminoglycan deposition in tissue, which avidly absorbs water [68].

Proopiomelanocortin (POMC) is a prohormone precursor protein expressed in the pituitary gland and is implicated in obesity. Some examples of endopeptidases are prohormone convertase 1 (PC1; also referred to as PC3 or PC1/3) and prohormone convertase 2 (PC2). These endopeptidases are responsible for converting POMC to MSH, which was described earlier. MSH is involved in appetite regulation. Traditionally, inactivating mutations in these endopeptidases were thought to result in obesity; however, recent research has suggested the importance of background factors and the altered production of other peptides in the development of obesity [69].

### 4.3. Hunger Cascade and the Brain–Gut Axis

Another crucial hormone in the hunger cascade is ghrelin, which has orexigenic action and stimulates appetite [70]. Ghrelin is primarily secreted by the gastric fundus [71]. The postprandial suppression of ghrelin has been demonstrated to be lower in obese individuals, which can explain the persistence of hunger in these individuals despite having just eaten a meal [72]. Leptin, another hormone, is secreted by adipose tissue and is a key energy regulator. Leptin acts on the arcuate nucleus of the hypothalamus which produces anorexigenic molecules. When energy stores are low, leptin levels decrease, resulting in increased appetite [73]. Obesity is characterized by hyperleptinemia, which should lead to decreased appetite, but obese individuals develop resistance to leptin, paradoxically resulting in increased appetite [74].

Recent research has demonstrated that signaling within the brain–gut–microbiome is implicated in the pathophysiology of obesity [75]. The gut microbiota is ever-evolving in a person’s lifetime and may be influenced by intrinsic or environmental factors. It is composed of trillions of microorganisms. The brain–gut axis mediates glucose homeostasis, appetite regulation, and gut motility [76]. Gut bacteria may affect the levels of crucial hormones such as glucagon-like peptide 1 (GLP-1) and ghrelin. There is circumstantial evidence that gut microbe composition may influence host eating behavior, but links between specific bacteria and hunger or satiety are still being researched [77].

### 4.4. Psychosocial Factors

Psychosocial factors may also contribute to the development of obesity. In children, poor mental health during the late childhood/early adolescence phase is associated with obesity development [78]. Depression has been associated with increased levels of inflammatory cytokines such as C-reactive protein, interleukin-6, and tumor necrosis factor-alpha (TNF-α) [79]. Similarly, obesity can result in a state of chronic low-grade inflammation and is characterized by overlapping inflammatory cytokines, including TNF-α and interleukin-6 [80]. The shared pathophysiology of both depression and obesity supports a bidirectional relationship [81].

### 4.5. Behavioral Factors

Behavioral factors also influence the development of obesity. Nutritional status can play a major role in weight gain [82]. Certain dietary habits such as snacking frequency, alcohol consumption, emotional eating, and a lack of fresh produce are risk factors for weight gain. The COVID-19 pandemic especially accentuated these risk factors as individuals were under self-quarantine and were more engaged in sedentary behaviors [83].

## 5. Management of Obesity

### 5.1. Obesity Stigma

Prior to discussing the management of obesity, it is crucial to recognize the stigma attached to this condition. Stigma towards obesity generally stems from the notion that it arises from the effects of an individual’s decision-making and failing to recognize the genetic and environmental factors that contribute. Because of this false assumption, individuals who are obese suffer from worse mental health, morbidity, and mortality [84,85]. Furthermore, socioeconomic status is linked with obesity as well. Particularly, highly processed foods are mass-produced, enabling them to be affordable, in contrast to fresh fruits and vegetables which may be more expensive. Obesity has been shown to affect underprivileged groups disproportionately, including minority and impoverished groups [86]. Obesity stigmatization is also seen in social media and entertainment, where characters who are obese may be portrayed with negative characteristics [87].

Concerningly, obesity stigma also extends to the healthcare setting as well. In a 2023 review article, patients with obesity reported feeling devalued during encounters with their physicians in which derogatory language or non-verbal behaviors were used. Particularly, there was a sentiment that they were being “talked down to” by healthcare professionals. In addition, patients reported their concerns not being taken seriously and simply being attributed to their obesity [88].

To address this stigma, healthcare professionals must ensure compassionate and respectful care. The notion that obesity is solely attributed to an individual’s volitional control must be dismissed. A person-centered approach focused on shared decision-making, discussion, and evidence-based guidelines is paramount in patients receiving quality and timely access to healthcare.

### 5.2. Lifestyle Interventions

Behavioral modification, namely exercise, is a cornerstone of obesity management. Exercise can prevent weight gain, result in weight loss, and can prevent weight regain after loss. Increased energy expenditure creates a more negative energy balance, resulting in weight loss [89]. Guidelines have recommended 150 to 300 min of moderate physical activity weekly [90]. A 2024 meta-analysis demonstrated that body weight decreased linearly with an increasing duration of aerobic exercise up to 300 min per week. The same relationship was also observed with waist circumference [91]. The use of GLP-1 receptor agonists, described in detail below, can result in weight loss as well. Weight regain can occur, however, in patients using GLP-1 receptor agonists after they stop taking them. A 2021 study demonstrated that compared to GLP-1 receptor agonist use alone, GLP-1 use in conjunction with moderate-to-vigorous intensity exercise can lead to improved healthy weight loss maintenance [92].

Dietary changes can also aid weight management. Caloric-restricted diets rich in fiber and lower in carbohydrates are thought to improve metabolic health and glucose homeostasis and be valuable in obesity management [93]. Interestingly, a low-carbohydrate diet has been shown to result in greater energy expenditure, which may assist with weight loss [94]. Time-restricted fasting, which includes intermittent fasting, was compared to consistent meal timing in a 2020 randomized control trial. It was determined that time-restricted fasting did not confer superior weight loss.

Certain diets have become popular over the years. For instance, the ketogenic, or keto, diet has risen in usage. In the ketogenic diet, carbohydrate intake is minimal. Specifically, individuals intake one gram per kilogram of protein, 10–15 g of carbohydrates, and the remaining calories from fats [95]. Physiologically, in these circumstances, insulin secretion is reduced due to the low levels of carbohydrates, glycogen stores are depleted, and stored fat is broken down into free fatty acids and ketones. The exact weight loss mechanism in a ketogenic diet is unclear. Hypotheses include increased gluconeogenesis, a highly expensive metabolic process, to provide energy for the brain. The ketogenic diet can also lead to decreased appetite. Notably, recent studies have demonstrated that the adoption of a ketogenic diet can increase low-density lipoprotein cholesterol levels [96].

The paleolithic, or paleo, diet has also risen in popularity in recent years. This diet restricts grains, dairy, and refined foods. The diet prioritizes grass-fed meats, vegetables, fruits, and nuts [97]. This low-carbohydrate diet can help promote weight loss, likely in similar ways to the ketogenic diet described above [98].

The sirtfood diet is relatively new and targets sirtuins (SIRTs), which are enzymes thought to play a role in calorie restriction, autophagy, cell proliferation, and inflammation [99]. Foods that activate SIRTs are thought to prevent metabolic and inflammatory disease. Polyphenols are examples of SIRT activators and are found in cereals, fruits, and vegetables [100]. These sirtfoods are thought to increase metabolism and decrease inflammation, resulting in weight loss.

Another popular diet is the Mediterranean diet, which centers around high fat intake, low carbohydrate intake, moderate-to-high fish consumption, and the low consumption of red meat [101]. It is routinely recommended by healthcare professionals. Epidemiological studies have demonstrated that those who adhere to the Mediterranean diet experience lower risks of cardiovascular disease [102]. Studies have shown that when energy-restricted (daily caloric intake is below requirements), the Mediterranean diet resulted in significant weight loss and a decrease in waist circumference [103]. This is likely mediated through early satiety and the overall decrease in caloric intake. A summary of the aforementioned diets is in Table 2.

### 5.3. Medical Intervention

#### 5.3.1. Earlier Medications

The major tenet of obesity management is lifestyle intervention, consisting of dietary changes and physical activity, as already mentioned. However, individuals have difficulty maintaining the weight loss achieved by these lifestyle interventions, with around 95% of people regaining lost weight within five years [104]. In these individuals, anti-obesity medications (AOMs) play a role and can lead to weight loss and the management of comorbidities [105].

Phentermine, a sympathomimetic agent, was one of the earliest Food and Drug Administration (FDA)-approved weight loss medications. It works by increasing the release of norepinephrine, serotonin, and dopamine and is thought to activate receptors leading to decreased food intake. There were concerns regarding its addiction potential due to its structural similarity to amphetamines. In the 1990s, phentermine was combined with fenfluramine and sold as “Phen-Fen;” however, it became less favored due to fenfluramine causing cardiac valvulopathies [106]. The majority of adverse reactions from phentermine are mild, including dry mouth, palpitations, and insomnia [107]. Notably, phentermine is also sold combined with topiramate, a carbonic anhydrase inhibitor, which promotes weight loss through taste aversion.

Orlistat is another AOM that inhibits pancreatic and gastric lipases, preventing triglyceride hydrolysis and hence the absorption of free fatty acids [108]. Given its mechanism of action, the most common adverse effect of orlistat is steatorrhea. It can also cause abdominal pain and diarrhea [109]. It can generally result in around 10% of total weight loss [110].

Naltrexone, an opioid receptor antagonist, can be paired with bupropion, a dopamine and norepinephrine reuptake inhibitor, to produce weight loss effects in adults. When coupled with intensive behavioral modification, naltrexone/bupropion was found to decrease body weight by 9.3% over 56 weeks [111]. Its long-term cardiovascular safety is under review [1].

#### 5.3.2. Recent Medications

GLP-1 receptor agonists have grown in popularity in recent years due to their potent weight loss effects. In 2015, patients with obesity were enrolled in a study, assigning them to either a once-daily subcutaneous liraglutide (a GLP-1 peptide receptor agonist) treatment group (3 mg once daily) or placebo. Over 56 weeks, the liraglutide group lost 8% body weight [112]. Further, in a major study in 2021, 1961 adults with obesity were randomly assigned either once-weekly subcutaneous semaglutide (another GLP-1 peptide receptor agonist) or placebo, in addition to lifestyle intervention. Over a 68-week period, the semaglutide group had a mean decrease in body weight of 14.9%, compared to a 2.4% decrease in the placebo group [113]. Common adverse effects from semaglutide included nausea and diarrhea. Subcutaneous semaglutide has also been linked to dysesthesia, and the effect may be dose-dependent [114]. In another trial, over a 72-week period, 4.9% of individuals on weekly 2.4 mg semaglutide experienced dysesthesia [115]. As highlighted by Chopra et al., semaglutide has implications beyond simply weight loss. It can modulate overall inflammatory levels in the body, affect endothelial function, and exert an influence on the cardiovascular risk profile [116]. These relationships are detailed later in Obesity and Cardiovascular Diseases and Risk Factors.

Semaglutide is also available in an oral formulation. In a 2023 study, patients were enrolled in treatment groups with 50 mg oral semaglutide daily vs. placebo. Over a 68-week period, patients taking oral semaglutide had a 15.1% decrease in body weight. The most common adverse effects included nausea, constipation, diarrhea, vomiting, decreased appetite, and dyspepsia [117]. A head-to-head study has not been performed yet comparing oral and subcutaneous semaglutide. Oral semaglutide is absorbed via the digestive system, while the injection is delivered subcutaneously. Doses with oral semaglutide are greater, as less of the medicine is actually absorbed into circulation. To promote absorption, oral semaglutide is recommended to be taken on an empty stomach first thing in the morning, 30 min prior to consuming any food or drink [118].

In 2025, another study was performed which enrolled patients in either a treatment group with 25 mg oral semaglutide daily or placebo. Over a 64-week period, the semaglutide group had a decrease in weight of 13.6%. While the rate of gastrointestinal side effects was similar to that in a previous trial involving 50 mg oral semaglutide daily, there was a lower rate of dysesthesia with 25 mg oral semaglutide daily [119].

Tirzepatide, another medication, is a once-weekly subcutaneous injection that acts on both the GLP-1 peptide receptor and the glucose-dependent insulinotropic polypeptide (GIP) receptor. In a 2022 study, over a 72-week period, tirzepatide resulted in a 15% decrease in weight, 19.5% decrease in weight, and 20.9% decrease in weight, with the 5 mg weekly, 10 mg weekly, and 15 mg weekly doses, respectively. Similarly to other medications like semaglutide, the most common adverse effects with tirzepatide are gastrointestinal-related and include nausea and diarrhea [120].

Interestingly, the incidence of side effects with these GLP-1 medications may be attributed to genetic differences. A 2026 genome-wide association study identified the linkage of the *GLP1R* locus (which encodes the GLP1 receptor) to differential side effects. Certain single-nucleotide polymorphisms may make individuals more prone to adverse effects like nausea and vomiting [121]. In addition, recent research studies suggest that GLP-1 therapies may result in muscle-based losses. However, more research is required to characterize how these losses functionally impact individuals [122].

While GLP-1 peptide receptor agonists offer promising weight loss effects, individuals regain body weight once the agent is discontinued. In a 2026 systematic review, individuals who discontinued semaglutide or tirzepatide returned to their baseline weight within 1.5 years, on average [123]. A recent preliminary study from the University of Alabama at Birmingham suggests that it might be possible to prevent weight regain after GLP-1 peptide receptor agonist discontinuation. In a preclinical study with rodents, researchers assessed the extent of weight regain after GLP-1 receptor agonist discontinuation. Mice were rendered obese using a high-fat diet. Mice were subsequently treated with semaglutide, and then GLP-1 was discontinued. After discontinuation, mice were either treated with a novel inhibitor of thioredoxin-interacting protein (TXNIP) or not. TXNIP is a protein implicated in beta-cell loss and pancreatic islet cell dysfunction. The group of mice that were treated with this novel agent had a significantly lower weight rebound. While further research must be conducted on this novel agent, it is a promising development in obesity management [124]. Table 3 summarizes the FDA-approved therapies for weight loss.

#### 5.3.3. Medical Therapies in the Pipeline

Retatrutide is a subcutaneous AOM that is still under clinical trial. It is unique in that it is a GLP-1, GIP, and glucagon (GCG) agonist. In a 2023 study, participants with obesity were randomly assigned to retatrutide treatment groups versus placebo. Retatrutide resulted in weight loss up to 17.5% over a 48-week period. The most frequent adverse effects were gastrointestinal (nausea, vomiting, diarrhea, constipation). It is presumed that GCG agonism allows for alterations to energy intake and expenditure that aid in weight loss [127].

Orforglipron is an oral AOM also under clinical development. It is a nonpeptide GLP-1 receptor agonist. In a 2025 study, participants with obesity were randomly assigned to orforglipron treatment groups versus placebo. At its maximum dose, the novel agent resulted in an 11.2% decrease in body weight. Orforglipron also led to clinically significant decreases in systolic blood pressure, triglycerides, and non-HDL cholesterol [128].

Another AOM under clinical trial is cagrilintide–semaglutide. It combines semaglutide, described above, with cagrilintide, an amylin analog shown to reduce food intake and promote weight loss. After a 68-week period, cagrilintide–semaglutide administered subcutaneously at a dose of 2.4 mg each once weekly demonstrated a 13.7% decrease in body weight. This AOM also demonstrated improved glycemic control in enrolled participants. The most common adverse effects experienced were gastrointestinal symptoms but only resulted in discontinuation in around 4.8% of participants [129]. Exciting combinations of different types of agonists with other substances and analogs currently in various stages of development promise a plethora of newer AOMs for managing obesity.

#### 5.3.4. Genetic Obesity Treatment

As mentioned earlier, setmelanotide is an MC4R agonist that helps increase satiety in patients with POMC deficiency, leptin receptor (LEPR) deficiency, or Bardet–Biedl syndrome (BBS), a rare autosomal recessive genetic disorder characterized by obesity, among other clinical manifestations. A 2025 study examined the role of setmelanotide in patients with POMC deficiency, LEPR deficiency or BBS. Setmelanotide was administered once daily subcutaneously for 52 weeks. The mean change in BMI from baseline to week 52 was −18%, highlighting the role it may play in managing obesity [130].

### 5.4. Surgical Intervention

Bariatric surgery is a weight loss intervention that can help individuals lose about 25–30% of total weight and is the most effective weight loss intervention. Most patients experience success after their surgery, but it is generally regarded as an option when conservative measures fail and is reserved for individuals with severe obesity [131,132]. Surgical candidates must have either a BMI greater than 40 kg/m^2^ or a BMI greater than 35 kg/m^2^ in conjunction with at least one obesity-related disease [1]. Common surgical techniques include the Roux-en-Y gastric bypass, gastric sleeve resection, and adjustable gastric banding. Nutritional and mental health evaluation precedes surgical intervention. Metabolic surgery confers an improved risk of mortality as well [133]. Furthermore, obesity is a chronic medical condition. While pharmacologic agents such as GLP-1 receptor agonists may result in weight loss, to sustain this weight reduction, individuals may be required to take these medications for life, which can be costly. Metabolic bariatric surgery may be less expensive in the long term [131]. Surgical intervention leads to malabsorption, mechanical restriction, and hormonal changes that aid in weight loss [71,134]. Caloric restriction is thought to play a role in initial post-surgical weight loss. Interestingly, post-surgically, patients may even experience changes in food preference and have reduced preference for sweet or high-calorie foods [135]. Individuals also experience higher levels of GLP-1 release post-surgically, which promote satiety. Peptide tyrosine, or peptide YY, is a polypeptide released by L-cells in the ileum, colon, and brain. Post-surgically, patients have higher levels of peptide YY, resulting in satiety and decreased food intake [136]. Complications or risks with bariatric surgery include deep vein thrombosis, post-surgical infection, nutritional deficiencies, and dumping syndrome [137]. Dumping syndrome is caused by rapid gastric emptying and the delivery of consumed intake to the small intestine. It is characterized by symptoms such as diarrhea, nausea, bloating, and flushing [138].

## 6. Obesity and Cardiovascular Diseases and Risk Factors

**Figure 5:**
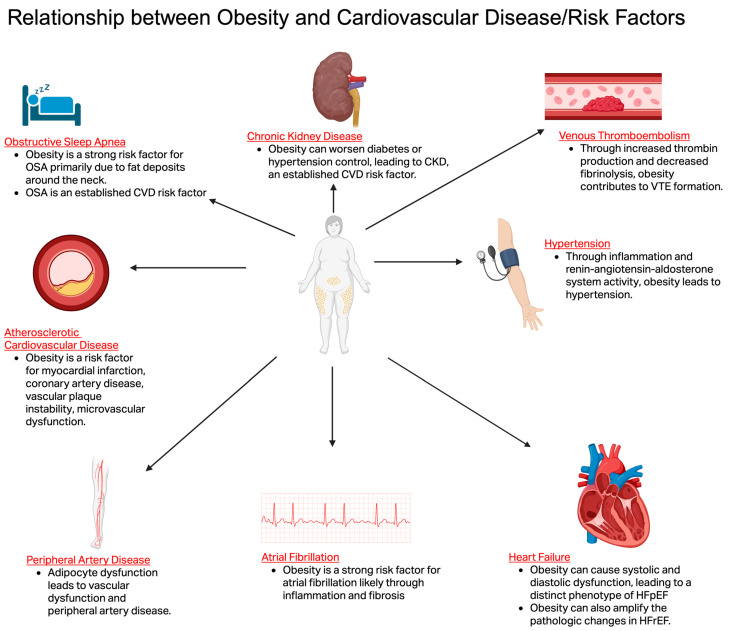
Relationship between obesity and cardiovascular disease and associated risk factors. CKD: chronic kidney disease; CVD: cardiovascular disease; HFpEF: heart failure with preserved ejection fraction; HFrEF: heart failure with reduced ejection fraction; OSA: obstructive sleep apnea; VTE: venous thromboembolism Illustration courtesy of Yaser Ahmad MD.

Figure 5 demonstrates the relationship between obesity and cardiovascular diseases/associated risk factors.

### 6.1. Atherosclerotic Cardiovascular Disease (Ischemic Heart Disease, Myocardial Infarction, Stroke, Peripheral Vascular Disease)

**Figure 6:**
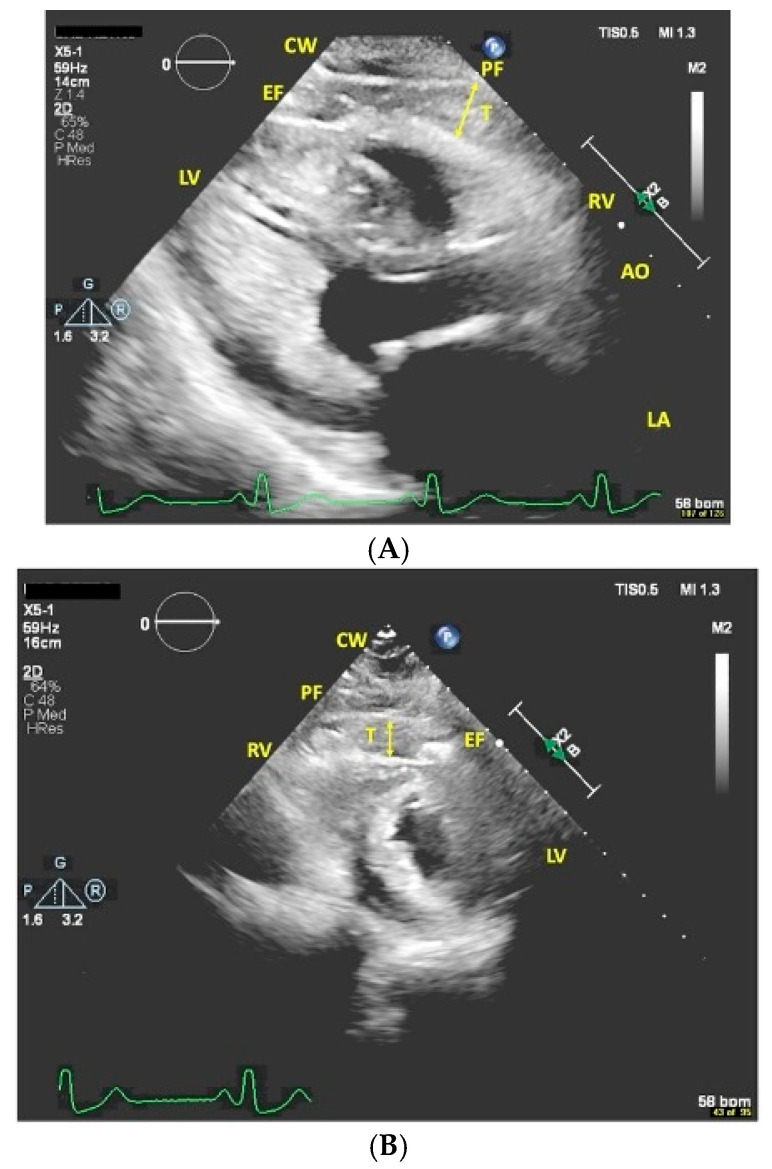
(**A**): Two-dimensional transthoracic echocardiographic assessment of epicardial adipose tissue (EF). Maximal thickness (T) of EF is 13 mm. Parasternal long-axis view. AO: Aorta; CW: Chest wall; LA: Left atrium; LV: Left ventricle; RV: Right ventricle; PF: Pericardial fat pad. Figure courtesy of Navin Nanda MD. (**B**): Two-dimensional transthoracic echocardiographic assessment of epicardial adipose tissue (EF). Maximal thickness (T) of EF is 14 mm. Parasternal short-axis view. Abbreviations as in Figure 6A. Figure courtesy of Navin Nanda MD.

There is a close relationship between obesity and atherosclerotic cardiovascular disease. The relationship between obesity and cardiovascular disease has been explored extensively in the past. For instance, the Framingham Heart Study has shown that being overweight or obese, as determined by BMI, is associated with increased cardiovascular disease risk [139]. Yusuf et al. demonstrated in the INTERHEART study that markers of abdominal adiposity were stronger risk factors for myocardial infarction than BMI [140]. VAT can specially create an inflammatory milieu associated with the development of atherosclerosis. Another major mediator of this relationship is EAT, described earlier. It is a fat layer between the myocardial and visceral layers of the epicardium, which expands with increasing obesity. As detailed in a review by Ahmad et al., increased EAT thickness is associated with obstructive coronary artery disease and even vascular plaque instability, predisposing one to acute coronary syndrome [141]. In addition, EAT is linked with coronary microvascular dysfunction [142]. EAT is contiguous with the myocardium as well as the adventitia of coronary arteries, which has been proposed as a trigger for atherosclerotic disease through the transmission of inflammatory mediators [143]. EAT can be routinely measured with two-dimensional transthoracic echocardiogram; Figure 6A,B demonstrate this. Aside from cardiovascular disease, as detailed by Manikandan et al. along with Wasan et al., increased EAT thickness is also associated with a host of other disease processes including but not limited to obstructive sleep apnea, rheumatoid arthritis, and systemic lupus erythematosus [53,144].

In addition, studies have shown that obesity is linked to peripheral artery disease (PAD). Excess adipose tissue accumulation leads to adipocyte dysfunction, systemic vascular dysfunction, and eventually atherosclerotic disease, leading to PAD [145]. Moreover, obesity is an established risk factor for stroke through the development of T2DM, hypertension, atrial fibrillation, and early atherosclerosis. Obesity is marked by insulin resistance, which can result in the onset of T2DM. Adipose-derived angiotensinogen and impaired nitric oxide production can lead to worsened hypertension. Obesity is also linked with platelet aggregation and overall hypercoagulability, likely secondary to impaired insulin sensitivity [146].

AOMs, specifically GLP-1 receptor agonists, have been associated with pleiotropic effects beyond just weight loss. It is presumed that these agents reduce inflammatory cytokines and plaque formation. In a landmark 2023 study, participants were enrolled in either a group subcutaneously injected with 2.4 mg semaglutide weekly or a placebo group. The primary endpoint for this study was a composite of cardiovascular death, nonfatal myocardial infarction (MI), and nonfatal stroke. With a mean follow-up time of 39.8 months, semaglutide was found to be superior to placebo in reducing the incidence of death from cardiovascular complications, nonfatal MI, or nonfatal stroke. In addition to anti-inflammatory effects, GLP-1 receptor agonists reduce the volume of ectopic adipose tissue that contributes to atherosclerotic disease. Also, these agents increase insulin sensitivity. In this study, GLP-1 receptor agonists were associated with improved lipid profiles, decreased levels of C-reactive protein, decreased waist circumference, and improved glycemic control [147].

### 6.2. Atrial Fibrillation

Atrial fibrillation (AF) is one of the most common arrythmias in the world and affects 60 million people worldwide. Risk factors for the development of AF include age, hypertension, T2DM, genetic factors, and obesity. After hypertension, obesity is thought to be the strongest risk factor and may explain up to 20% of all AF cases [148,149]. Incremental increases in BMI have been associated with an increased risk of AF [150]. Beyond the development of AF, obesity is also implicated in its persistence and severity [151]. Mechanistically, left atrial enlargement in obese individuals predisposes them to the development of AF [152]. Obesity also stimulates renin–angiotensin–aldosterone system (RAAS) activity, leading to increased sympathetic tone, atrial stretch, and vascular resistance, all of which increase the likelihood of AF development [153]. In addition, EAT is thought to possess arrhythmogenic properties and can predispose obese individuals to AF by way of inflammation, fibrosis, and atrial electrical remodeling [154]. Interestingly, even after catheter ablation for AF, obese individuals demonstrate higher levels of recurrence, emphasizing the need for metabolic optimization. A 2025 study concluded that semaglutide therapy is associated with a reduced risk of AF recurrence in obese patients undergoing catheter ablation [155]. It is thought that GLP-1 receptor agonists result in weight reduction and increased insulin sensitivity, which decrease inflammatory activity.

### 6.3. Heart Failure with Preserved Ejection Fraction–Obesity Phenotype

Greater than 80% of patients with heart failure with preserved ejection fraction (HFpEF) are overweight or obese. It is hypothesized that increased adipose tissue results in more inflammation, insulin resistance and hypertension, which impair both systolic and diastolic dysfunction [156]. Myocardial biopsies of individuals with obesity have demonstrated sarcomere disruption, lipid droplet accumulation, and disruptions in fatty acid processing and oxidation, all proportional to the degree of obesity [157]. These pathophysiologic changes affect the systolic and diastolic function of the heart and produce a distinct obesity phenotype of HFpEF. Interestingly, increased BMI may predispose women more than men to HFpEF [158]. In 2023, a landmark study was conducted, which enrolled patients with HFpEF and obesity to either a group receiving a subcutaneous injection of 2.4 mg semaglutide weekly or a placebo group. They used a standardized questionnaire to assess symptoms pertaining to heart failure. Individuals in the semaglutide treatment group had, on average, a 13.3% decrease in body weight compared to 2.6% with placebo. By the end of the study period, participants in the semaglutide treatment group had decreased symptom burden and increased six-minute walk distance [159].

### 6.4. Heart Failure with Reduced Ejection Fraction

While the relationship between obesity and HFpEF is well established, the link between obesity and heart failure with reduced ejection fraction (HFrEF) has been receiving more attention. Obesity is a disease modifier and can amplify the pathophysiologic changes seen in HFrEF. Increased body weight leads to the neurohormonal activation of the renin–angiotensin–aldosterone system (RAAS), resulting in myocardial fibrosis and adverse ventricular remodeling [160]. There are also hemodynamic alterations in the obese state, namely increased plasma volume and venous return. This can lead to increased left- and right-sided filling pressures, eventually causing systolic dysfunction [161]. Current data does not demonstrate a significant benefit of GLP-1 receptor agonists in HFrEF. However, research on this topic is ongoing, and future studies focused on patients with obesity are needed [162]. As detailed by Spanova et al. in a review on obesity, bariatric surgery in patients with HFrEF has been associated with a decreased frequency of hospitalizations for HFrEF exacerbations [163].

### 6.5. Hypertension

Individuals with obesity may be at risk for essential or resistant hypertension. The link between obesity and increased blood pressure is likely due to increased inflammation, RAAS activity, and insulin resistance [164]. In animal models, GLP-1 receptor engagement activates brainstem catecholamine neurons, which attenuates sympathetic activity, thereby decreasing blood pressure. A meta-analysis was published in 2022 evaluating the effects of liraglutide, a GLP-1 receptor agonist in various trials. It was noted to decrease systolic blood pressure on average by 3.07 mmHg and diastolic blood pressure by 1.01 mmHg [165]. A 2.4 mg once-weekly subcutaneous semaglutide injection was found to decrease systolic blood pressure by 6.16 mmHg over a 68-week period of time [113]. A meta-analysis including six randomized control trials also produced similar conclusions, stating that once-weekly semaglutide injections provided an average decrease in systolic blood pressure of 4.83 mmHg [166].

### 6.6. Venous Thromboembolism

In a 2008 meta-analysis, 63,552 patients were included to study cardiovascular risk factors leading to venous thromboembolism (VTE) formation. Obesity was found to be the strongest risk factor in comparison to other traditional cardiovascular risk factors such as hypertension, T2DM, tobacco use, and hyperlipidemia. It is thought that central adiposity is associated with increased thrombin production, decreased fibrinolysis, and immobility, which can all contribute to VTE formation.

### 6.7. Obstructive Sleep Apnea

Obstructive sleep apnea (OSA) has well-established links to cardiovascular disease. Obesity is a risk factor for OSA [167]. Repeated apneic episodes at night, sleep fragmentation, and intermittent hypoxia can put one at risk for cardiovascular diseases such as hypertension and heart failure [168]. Given that obesity is one of the strongest risk factors for OSA, target intervention around body weight is imperative. Weight loss can lead to less hypoxia and fewer apneic events overnight [169]. A 2024 study recruited participants with moderate-to-severe OSA and obesity. Patients receiving positive airway pressure (PAP) treatment and also those not receiving PAP treatment were randomized to either tirzepatide or placebo treatment groups. The tirzepatide treatment groups were associated with lesser hypoxic burden, improved blood pressure, and improved patient-related sleep outcomes such as decreased sleep disturbance or impairment [170].

### 6.8. Chronic Kidney Disease

Obesity is also a risk factor for the development and progression of chronic kidney disease (CKD). Obesity has a strong link to T2DM as well as hypertension, which can predispose one to CKD [171]. Adipocytes in obesity may also produce adipokines that contribute to CKD. Particularly, adipokines can influence inflammation, leading to a decreased glomerular filtration rate. They also exert effects on tubular networks and can cause albuminuria [172]. Diet and physical activity are recommended as the cornerstone to obesity management in individuals with CKD. However, there is also a role for incretin mimetic agents such as GLP-1 receptor agonists. A 2024 study examined the effects of semaglutide on kidney-related outcomes. It recruited patients with T2DM and CKD and randomly assigned them to an injectable semaglutide or placebo treatment group. The semaglutide group had a statistically significant lower incidence of the primary composite outcome consisting of kidney failure, a 50% reduction in the glomerular filtration rate from baseline, and death from a kidney or cardiovascular cause [173].

### 6.9. Type 2 Diabetes Mellitus

T2DM is an established risk factor for cardiovascular disease. Adults with diabetes have a 2–4 times increased cardiovascular risk compared to adults without diabetes. It is associated with coronary disease, heart failure, and peripheral vascular disease [174]. Previous studies have shown a demonstrable risk reduction in cardiovascular morbidity and mortality in patients with T2DM treated with GLP-1 receptor agonists. GLP-1 receptor agonists lower blood pressure and also lipid levels, which may explain this decrease in major adverse cardiovascular events [175]. GLP-1 receptor agonists may also decrease vulnerable plaque formation, leading to fewer cardiovascular events [176].

## 7. Conclusions

In summary, obesity is a chronic condition, characterized by a complex interplay between genetic, environmental, behavioral, and physiologic factors. There are a variety of indices used to classify obesity, each with its advantages and disadvantages. Imaging modalities such as computed tomography and dual-energy X-ray absorptiometry may also be used, but they are less practical. Point-of-care ultrasound is a powerful and practical imaging modality for assessing body fat, although it has limitations including operator dependence, variable measurement landmarks, and a lack of standardized reference values. There are both established and up-and-coming anti-obesity medications that have been shown to be effective. While these medications are becoming increasingly popular, dietary and lifestyle modifications remain key for managing obesity as well. There is also utility in surgical intervention for select patients. There is a strong link between obesity and cardiovascular diseases and associated risk factors. These include atherosclerotic disease, heart failure, hypertension, T2DM and atrial fibrillation. Recently developed anti-obesity medications show promise for cardiovascular benefit in addition to weight loss, and future anti-obesity work should distinguish between medications with evidence primarily for weight loss versus for cardiovascular benefit.

## Figures and Tables

**Table 2:** Summary of dietary trends.

Diet	Characteristics	Weight Loss Mechanism
Ketogenic diet	One gram per kilogram of protein, 10–15 g of carbohydrates, and the remaining calories from fats.	Increased gluconeogenesis, which is metabolically expensive; decreased hunger; decreased overall caloric intake.
Paleolithic diet	Grass-fed meats, vegetables, fruits, and nuts. Individual variation exists.	Early satiety, decreased overall caloric intake.
Sirtfood diet	Various fruits and vegetables high in polyphenols.	Increased metabolism and decreased inflammation by activating sirtuins.
Mediterranean diet	High fat, low carbohydrates, moderate-to-high fish intake, low red meat consumption.	Early satiety, decreased overall caloric intake.

**Table 3:** FDA-approved therapies for weight loss.

Drug Name	Mechanism of Action	Method of Administration	Dosing	Efficacy	Common Side Effects
1. Orlistat	Pancreatic and gastric lipase inhibitor	Oral (during or 1 h after a fat-containing meal)	60 mg three times daily or 120 mg three times daily.	9.8% weight loss at 6 months (120 mg dose) [125].	Diarrhea, abdominal pain, steatorrhea
2. Phentermine/Topiramate	Increases release of norepinephrine/taste aversion	Oral (once daily with morning meal)	3.75/23 mg, 7.5/46 mg (lowest treatment dose), 11.25/69 mg or 15/92 mg.	~8.3% weight loss at week 56 [126].	Dizziness, paresthesia
3. Bupropion-naltrexone	Opioid receptor antagonist + dopamine and norepinephrine reuptake inhibitor	Oral	Starting dose: one tablet (naltrexone 8 mg/bupropion 90 mg) once daily. Increase by weekly intervals up to two tablets twice daily.	9.3% weight loss when combined with intensive behavior modification after 56 weeks [111].	Nausea, headache, constipation
4. Liraglutide	Glucagon-like peptide 1 receptor agonist	Subcutaneous	Starting dose 0.6 mg/day. Increase at weekly intervals by 0.6 mg/day. Target dose is 3 mg/day.	8.0% weight loss over 56 weeks (3.0 mg daily dose) [112].	Nausea, vomiting, cholelithiasis, cholecystitis
5. Semaglutide (subcutaneous)	Glucagon-like peptide 1 receptor agonist	Subcutaneous injection	Weekly titration: 0.25 mg once weekly, 0.50 mg once weekly, 1 mg once weekly, 1.7 mg once weekly, 2.4 mg once weekly (maintenance).	−14.9% weight loss at week 68 (2.4 mg dose) [113].	Nausea, diarrhea, pancreatitis
6. Semaglutide (oral)	Glucagon-like peptide 1 receptor agonist	Oral	Monthly titration: 1.5 mg once daily, 4 mg once daily, 9 mg once daily, 25 mg once daily (maintenance).	At week 64, 13.6% decrease in weight with 25 mg dose [119].	Nausea, diarrhea, constipation, dyspepsia, lack of appetite
7. Tirzepatide	Glucagon-like peptide 1 receptor and glucose-dependent insulinotropic polypeptide agonist	Subcutaneous injection	Weekly titration: 2.5 mg once weekly and increase by 2.5 mg/week increments to a maximum of 15 mg weekly.	At week 72, 15% decrease with 5 mg dose, 19.5% decrease with 10 mg dose, and 20% decrease with 15 mg dose.	Nausea, diarrhea, constipation

## Data Availability

No new data were created or analyzed in this study.
